# Secondary Metabolites Predict Diazotrophic Cyanobacteria: A Model-Based Cheminformatic Approach

**DOI:** 10.3390/metabo15090562

**Published:** 2025-08-22

**Authors:** James Young, Taufiq Nawaz, Liping Gu, Ruanbao Zhou

**Affiliations:** Department of Biology and Microbiology, College of Natural Sciences, South Dakota State University, Brookings, SD 57007, USA; james.young@jacks.sdstate.edu (J.Y.); taufiq.nawaz@jacks.sdstate.edu (T.N.);

**Keywords:** secondary metabolites, chemical similarity, diazotrophic cyanobacteria, nitrogen fixation, predictive modeling, biomarker, cheminformatics

## Abstract

**Background:** Nitrogen fixation (diazotrophy) is a desirable trait present in some cyanobacteria with potential applications in sustainable agriculture and chemical feedstock production. This study discovers a predictive relationship modeled between secondary metabolites and diazotrophic cyanobacteria by leveraging chemical structure similarity to identify diazotrophic strains. **Methods:** An algorithm was developed using chemical fingerprint similarity of metabolites curated from CyanoMetDB and evaluated with leave-one-out cross-validation on 133 manually labeled metabolites. **Results:** The model demonstrated strong predictive performance, achieving 88% accuracy and a ROC-AUC of 0.96. We then applied this approach to prioritize likely diazotrophic strains among 1980 unlabeled metabolites and their associated organisms, providing a rank order of most likely undetected diazotrophic strains. Toxicity analysis showed that diazotrophic-associated metabolites show similar toxicity to non-diazotrophic metabolites in rats, with less toxicity in Daphnia magna, suggesting that these metabolites are not playing a defensive role. However, these metabolites did have relatively high nitrogen presence, and many were cyclic peptides, potentially serving as signaling molecules. **Conclusions:** This study underscores the potential of secondary metabolites in identifying diazotrophs, even when they may not be actively demonstrating diazotrophic physiology. Discovering more diazotrophic cyanobacteria has strong implications for advancing agricultural biotechnology towards the goal of self-fertilizing crops.

## 1. Introduction

Cyanobacteria, the focus organisms of this study, are ancient photosynthetic prokaryotes that have evolved to thrive in diverse environments under pressures ranging from extreme heat and cold to bioavailable nitrogen deprivation [1]. Cyanobacteria’s ability to convert atmospheric dinitrogen gas (N_2_) to bioavailable ammonia is also known as diazotrophy and is unique to prokaryotes [2]. Due to the coupling of diazotrophy with photosynthesis in cyanobacteria, they are a prime research focus for researchers aiming to transfer this self-fertilizing trait to cereal crops. There have been attempts to transfer nitrogen-fixing genetic complements to plant mitochondria [3] as well as into non-diazotrophic cyanobacteria [4,5] with limited success. The payoff of successfully transferring diazotrophy into cereal crops is both an environmentally and economically friendlier agricultural production system [6] that would lessen our dependency on the Haber-Bosch process for agricultural fertilizer.

In addition to their unique capabilities for both photosynthetic carbon (CO_2_) fixation and photosynthetic nitrogen fixation [7], cyanobacteria are also a rich source of secondary metabolites [8]. Secondary metabolites are chemicals produced by the organism that are not directly necessary for building new cells or creating energy but rather carry out roles such as antibacterial effects or protease inhibition, which can help the cell survive [9]. Prior study has focused on cyanobacterial secondary metabolites as potential drugs [9,10,11] and cosmetic additives [11] and shown that the habitat of the cyanobacteria is strongly associated with unique secondary metabolites.

We hypothesized diazotrophic cyanobacteria share distinctive secondary-metabolite structural signatures that can be recognized computationally and used to infer nitrogen-fixing capability. These structural differences may arise from selective pressures associated with diazotrophy, such as the need for nitrogen-rich scaffolds that serve as storage reservoirs or signaling molecules in nitrogen-deprived environments. The existing literature has used isotope labeling to elucidate gene clusters responsible for cyanobacterial secondary metabolite production when grown under non-diazotrophic conditions [12]; however, we have not found any literature that uses secondary metabolites as predictors for the diazotrophic capabilities of the cyanobacteria that produce them. Unlike traditional quantitative structure–activity relationship (QSAR) modeling, which links structure to the molecule’s own bioactivity, our approach predicts an organismal phenotype (diazotrophic or not) from the metabolites it produces, providing a phenotype-from-metabolite ‘twist’ on QSAR.

Across cyanobacteria, some non-ribosomal peptide synthetase/polyketide synthase biosynthetic gene cluster (NRPS/PKS BGC) families track phylogeny within particular clades, such as the CF-1 PKS family linked to heterocyst glycolipid biosynthesis in a heterocystous clade [13,14]. However, at the phylum scale, BGCs show complex histories with signatures of horizontal gene transfer (HGT) and recombination rather than simple vertical inheritance, with evidence including mobility elements and atypical dinucleotide composition [13]. Synthesizing this prior research, there is support for the hypothesis that diazotrophic cyanobacteria have distinctive and identifying chemotypes, even within the reality of high HGT of BGC overall in cyanobacteria [15].

There is also pre-existing evidence regarding the hypothesized roles as specialized signaling or storage molecules in diazotrophic cyanobacteria. *Nostoc punctiforme*, a diazotrophic and heterocystous cyanobacterium, secretes the cyclic secondary metabolite nostopeptolide (NRPS/PKS–derived), which acts as an autogenic hormogonium-repressing factor and shows concentration-dependent chemoattraction [16]. In regard to the hypothesis that these secondary metabolites could be nitrogen storage molecules, cyanophycin is a well-known example where nitrogen-rich amino acids are polymerized without the use of ribosomes. However, cyanophycin granules are more macro-level molecules, and we could not find the literature as of now on secondary metabolites playing a role in nitrogen storage or sharing, unlike inorganic forms of fixed nitrogen, which have been more widely reported in fixed-nitrogen sharing [17]. While the purpose of this paper is not to elucidate the molecular mechanism(s) of these potential metabolites’ roles, the existing literature seems to support a hypothesis of signaling more than storage.

The aim of this research is to use the fingerprint similarity of chemical structures through rcdk [18] to build a probability score that the metabolite came from a diazotrophic cyanobacteria. To enhance code reproducibility, generative AI was used to modularize and refine the R scripts that produce the analyses supporting our claims, with all outputs manually validated (more details in Section 2). Supporting the value of computational approaches in bioprospecting, recent reviews show that deep-learning pipelines now bridge genomes, metabolite structures, and bioactivity data, accelerating natural-product discovery [19]. An accurate model that allows prediction of whether diazotrophic cyanobacteria are in environmental samples based on secondary metabolites could speed up processing environmental samples by prioritizing study for samples with stronger diazotroph-associated metabolites. Moreover, there is potential for this approach to identify environmental samples with diazotrophic cyanobacteria that temporally regulate nitrogen fixation, which could otherwise have misleading negative results if relying solely on nitrogenase assays.

This study is a necessary step towards speeding up the discovery of diazotrophic cyanobacteria by using a metabolite-based approach and is analogous in its computational focus to recent studies predicting antiviral activity of cyanobacterial secondary metabolites using machine learning [20]. Discovering more unique diazotrophic cyanobacteria can bring the field closer to a long-standing goal of transferring this trait into cereal crops [21]. The importance of discovering new diazotrophic cyanobacteria lies in their diversity of strategies to balance oxygen-labile nitrogenase activity with photosynthesis. Some of the ways cyanobacteria are known to separate nitrogenase from oxygenic activity include temporally [22], spatially through differentiated cells like heterocysts [23,24], and spatially within the same cell [25]. Recent comprehensive reviews detail both the expanding diversity of cyanobacterial natural products [26] and the latest advances in exploiting diazotrophic cyanobacteria for sustainable agriculture [27], underscoring the timeliness of a metabolite-centered approach to diazotrophy prediction. Finding more strategies for reconciling the nitrogenase and oxygenic activity would contribute to a fuller understanding of the basic science and broaden the approaches available for transferring this trait and engineering self-fertilizing crops.

## 2. Materials and Methods

Data were curated from CyanoMetDB [28], specifically the Feb 2021 version available at https://zenodo.org/records/4562688 (accessed on 18 August 2025). SMILES strings were used as supplied with no special processing outside of their direct fingerprinting and subsequent analysis in the provided code. This version of CyanoMetDB contains 553 cyanobacterial strains (when counting by unique genus, species, and strain combinations) contributing 2114 metabolite entries, one metabolite of which was dropped due to a SMILES fingerprinting defect. Strains of cyanobacteria within the database were manually checked on UniProt [29] for having NifH, NifD, or NifK proteins associated with them as these are markers for nitrogen fixation. Strains having one or more of these proteins present in their proteome were marked FIX = “1” for nitrogen-fixing, and strains without any of these proteins were marked FIX = “0” for not nitrogen-fixing. If a strain did not have a sufficient proteome size (>100) and did not have any of the described Nif proteins, then it was not marked 0 or 1 and was left as unlabeled. This exclusion criteria left 492 observations containing unique genus sp. strain names as unlabeled (~90% of the 553 total CyanoMetDB unique named strains), leaving 61 strains with sufficient proteome coverage for reliable diazotrophy classification. Furthermore, 100 proteins on UniProt were used as the threshold for being marked as unlabeled to minimize the risk of labeling false negatives [25]. The rationale is that diazotrophy is a well-known attribute within the cyanobacterial research community, and *nif* genes and their proteins are often targeted for sequencing early in the strain characterization process. These genes are even targeted to the extent that they are used for phylogenetic purposes [30,31]. The risk of leaving strains and metabolites unlabeled is lower than that of a false negative and provides a second layer of validation, as seen in our results, where an unlabeled strain of *Schizothrix*, a genus known for diazotrophy, had one of the highest scores for being a likely diazotroph.

CyanoMetDB was filtered to have only chemicals associated with labeled strains to train and evaluate the model. The model was built in R by fingerprinting the SMILES using the rcdk package [18], which numerically represents its associated chemical structure. We specifically used the get.fingerprint() function of rcdk version 3.8.1. For the primary modeling, including leave-one-out cross-validation (LOOCV) and confusion matrix generation, we used the path-based standard fingerprint with a search depth of 30 and a bit length of 262,144. Additionally, we assessed alternate configurations (extended d15 262 k bits, PubChem 881 bits, graph 4096 bits, and MACCS 166 bits), all having generally similar performance. Model performance for each approach was evaluated using 10 random repeats of stratified 10-fold cross-validation to maintain the proportion of diazotrophic and non-diazotrophic strains within each fold.

Path-based d30 262 k fingerprints were selected for the primary analysis because of the balance they provide between comprehensive structural representation and computational efficiency. While PubChem fingerprints achieved slightly higher accuracy (90.5% vs. 86.8%), the path-based approach captures longer molecular pathways (up to 30 bonds) that better represent the complex cyclic peptide structures characteristic of diazotroph-associated metabolites. Additionally, the 262 k bit length provides sufficient resolution to distinguish structural nuances while maintaining reasonable computational requirements for leave-one-out cross-validation on our dataset.

The model studies by finding the most similar labeled metabolite fingerprint representation to the chemical you are querying, gives its similarity score (between 0 and 1) and the class of that database chemical. The formula for modeling the probability is conditional on whether the most similarly labeled metabolite was associated with a diazotrophic or non-diazotrophic cyanobacterium and is thoroughly described in Section 3. This algorithmic approach was evaluated using leave-one-out cross-validation (LOOCV) and 10 randomized repeats of 10-fold cross-validation. Analyses were conducted in R version 4.5.1 on Windows 11, using the following core packages: tidyverse 2.0.0, here 1.0.1, splitstackshape 1.4.8, rcdk 3.8.1, fingerprint 3.5.7, rmarkdown 2.29, factoextra 1.0.7, ggdendro 0.2.0, caret 7.0-1, pROC 1.18.5, yardstick 1.3.2, gains 1.2, ggpubr 0.6.1, and patchwork 1.3.1. All package versions, code, and results are available via the links provided in the Supplementary Materials section. Generative AI (OpenAI’s GPT-4 and Claude) was used specifically for code modularization, function optimization, and documentation enhancement. The AI assisted in restructuring existing analysis scripts into reusable modules and improving code readability. All AI-generated code was manually reviewed, tested against original results for equivalence, and validated by the authors before inclusion. The decision threshold for being predicted as a diazotrophic class was a final score over 0.5, derived by the equation in Section 3 (not just similarity), and the global threshold in this paper for statistical significance is *p* < 0.05.

## 3. Results

### 3.1. Design and Performance

**Figure 1:**
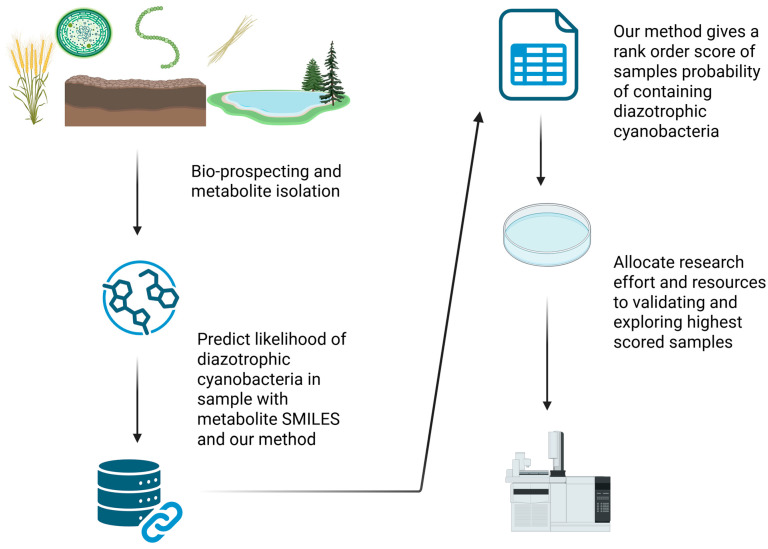
Practical application of this methodology in practice. Researchers may isolate cyanobacterial samples and their metabolites for many reasons, not always with an eye toward nitrogen fixation, and even when that is a focus, they may miss the temporal or otherwise regulated aspect of diazotrophic capability in single assays. Researchers may also have a hard time justifying the resources to thoroughly culture and assay all field samples depending on the volume of samples collected. However, with the collation of these metabolite results in resources like CyanoMetDB, the discovery of previously unknown diazotrophic strains could be sped up by this approach.

We constructed an algorithm to estimate the probability that a diazotrophic cyanobacterium would be in a sample based on detected metabolites. Our model uses chemical similarity between the unknown metabolite and the known diazotrophic and non-diazotroph associated metabolites. The algorithm was trained using metabolites from CyanoMetDB [28], which were manually labeled as diazotrophic or non-diazotrophic, as described in Section 2. A view of the secondary metabolite driven workflow and one pragmatic use case is shown below in Figure 1.

#### 3.1.1. Metabolite Diversity Represented

**Figure 2:**
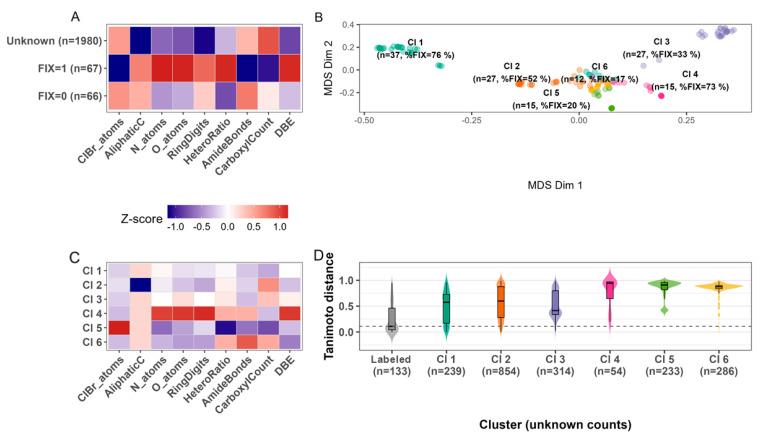
Structural descriptors discriminate FIX (FIX = 1 is diazotrophic, FIX = 0 is non-diazotrophic) activity and organize secondary metabolites into clusters. (**A**) Z-score heat map of nine structural descriptors for the labeled reference set (FIX = 1, n = 67; FIX = 0, n = 66) and the full set of unique unlabeled (“Unknown”, n = 1980) metabolites, where red means higher than the global average and blue means lower. (**B**) Multidimensional scaling (MDS) of Tanimoto distances for the 133 labeled metabolites. Unknowns are colored by the six clusters (Cl 1–Cl 6) obtained (color consistent with 2D). The size of each label gives the cluster size (n) and the percentage of FIX-positive standards within the cluster. (**C**) Cluster-level descriptor heat map (cluster medians with the same color scale as in 2A) highlighting chemical signatures that distinguish clusters. (**D**) Violin-and-box plots of intra-cluster Tanimoto distances for the unknown metabolites sorted into each cluster relative to their closest similarity labeled metabolite within that cluster, demonstrating chemical diversity. Together, the panels demonstrate that readily computed descriptors separate FIX = 1 from FIX = 0 metabolites and that unknowns occupy a range of chemical spaces, some with high similarity and some more novel relative to labeled metabolites.

Exploration of the chemical space confirmed that the nine simple descriptors selected (halogen content, heteroatom counts, ring architecture, hetero-to-carbon ratio, selected functional groups, and double bond equivalents) capture differences between diazotrophic (FIX = 1) and non-diazotrophic (FIX = 0) reference metabolites (Figure 2A). FIX positive standards were characterized by higher nitrogen content, a greater abundance of amide bonds and carboxyl groups, and lower halogenation. Importantly, the same descriptors were calculated for every structure in our 1980-member unknown set. These are aggregate representations of the classes’ intensity of those variables relative to the variable global average and are represented as a heatmap of the resulting z-scores. More consideration to these chemical descriptors will be given in the discussion.

Projecting all structures into a Tanimoto chemical distance space and partitioning the labeled metabolites by hierarchical clustering yielded clusters (Cl 1–Cl 6; Figure 2B–D). The proportion of FIX-labeled metabolites varies markedly among clusters (17–76%), providing a heuristic baseline of predictive ability of chemical structure to diazotrophic capabilities. We will expand on this predictive ability further below. Collectively, this analysis links readily computed chemical features of secondary metabolites associated with nitrogen fixation potential and supports the hypothesis that secondary metabolites are differentiated between diazotrophic and non-diazotrophic cyanobacteria due to the implications of the selective pressures and advantages of diazotrophic metabolism.

Of the 1729 secondary metabolites with a genus label present, 77% were observed in only a single genus, and 74% were in a single genus sp. strain. While broad chemotypes (like nitrogen-rich cyclic peptides) recur across genus, the exact metabolite structures are typically private to one lineage, consistent with strong vertical inheritance or limited horizontal transfer. Consequently, even our shallow nine-descriptor space captures a high degree of exclusivity, providing a mechanistic explanation for the cluster-level enrichment of diazotrophs (17–76% FIX = 1 in Cl 1–Cl 6, Figure 2B). These observations suggest that there is metabolic uniqueness across the CyanoMetDB and that structural fingerprints can encode lineage-specific biochemical signatures.

#### 3.1.2. The Similarity-Based Diazotrophic Prediction Algorithm

The algorithm for predicting the strength of diazotroph association likelihood is as follows:

(1) P(FIX = 1 | s) = 0.5 + 0.5 s (2 I_NN = 1 -1)

where the predicted probability of a metabolite being diazotroph associated (FIX = 1) is conditional on it having a chemical fingerprint similarity (s), measured by the Tanimoto approach, to a known diazotroph or non-diazotroph metabolite. The calculated probability is determined by both the magnitude of its Tanimoto similarity to its nearest neighbor (NN) and the FIX class of the NN (1 or 0). If I_NN (FIX class) = 1 is true, then (2 I_NN = 1 -1) = 1, or if I_NN = 0, (2 I_NN = 1 -1) = -1 by using true/false logic with the NN FIX class. An example of an unknown metabolite with 70% similarity to a known diazotroph-associated metabolite would be P(FIX = 1 | 0.7) = 0.5 + 0.5 (0.7) (2 (1)-1) = 0.85 and therefore predicted to be diazotroph associated. An example of an unknown metabolite with 70% similarity to a known non-diazotroph-associated metabolite would be P(FIX = 1 | 0.7) = 0.5 + 0.5 (0.7) (2 (0)-1) = 0.15 and therefore predicted to be non-diazotroph associated. The similarity score can be derived from various chemical fingerprinting approaches and still achieve high predictive performance, as demonstrated in Table 1. This approach is validated in both the leave-one-out cross-validation and 10 random repeats of 10-fold cross-validation presented in this paper. This approach is similar to other studies where nearest neighbor similarity was mapped to a probabilistic prediction of the activity of the observation of interest [32].

**Figure 3:**
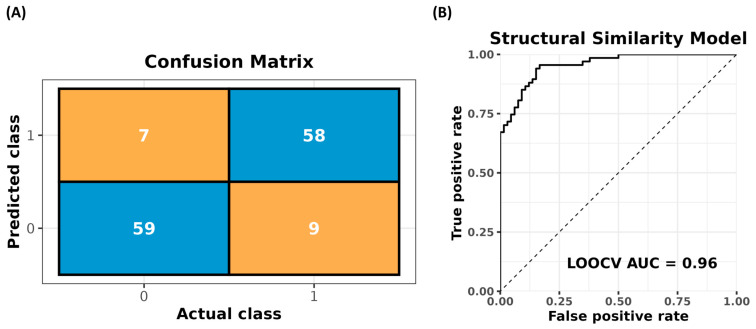
This approach has strong performance in predicting whether the producer of the secondary metabolite is a diazotrophic cyanobacterium or not. (**A**) The confusion matrix shows that this approach frequently correctly predicts whether a secondary metabolite is associated with diazotrophic (1) or non-diazotrophic (0) cyanobacteria on observations not seen in training during the leave-one-out cross-validation (LOOCV). Orange blocks are the counts of incorrect predictions in LOOCV, and blue are the counts of correct predictions. (**B**) The ROC-AUC of the holdouts is near a perfect score with a value of 0.96, further supporting the power of this approach’s rank-ordering.

Our structural similarity-based model predicted the correct class (diazotrophic or non-diazotrophic) with 88% accuracy (117 correct predictions/133 observations) and a receiver operating characteristic area under the curve (ROC-AUC) of 0.96 on leave-one-out cross-validation holdouts (133 manually labeled compounds total). These results are visualized below in Figure 3. If a person were to make the naive prediction that all chemicals represented the majority class (diazotrophic) in the training set, they would only be correct 50.3% of the time.

### 3.2. Top Unknown Strains and Metabolites for Exploration

**Figure 4:**
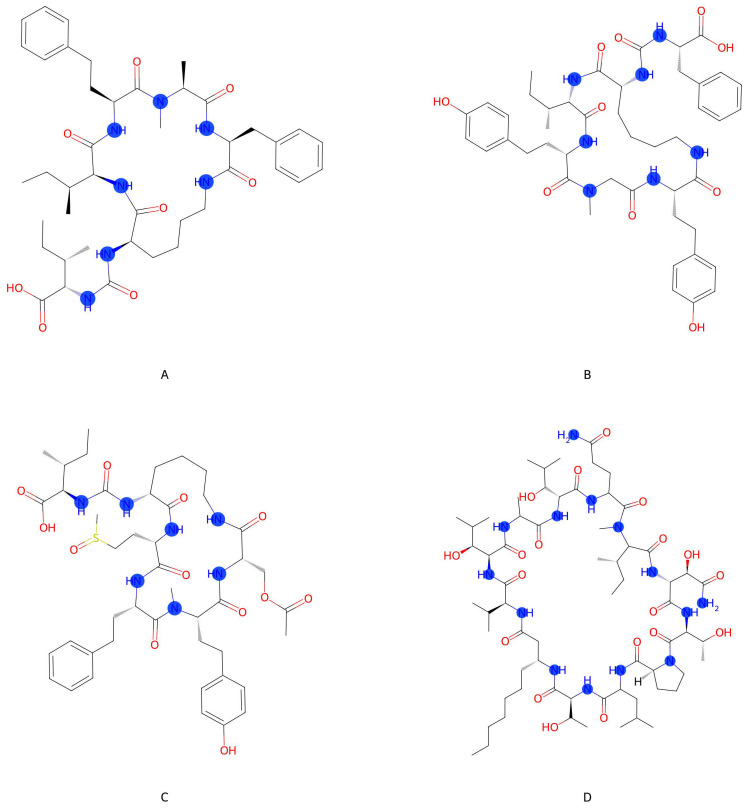
Representing selected secondary metabolites of interest from Table 3, where the blue dots emphasize nitrogen sites in the compound and all other aspects follow conventional chemical visualization. These examples are all nitrogen-rich cyclic peptides, but not all cyclic peptides are highly predictive of diazotrophs. These cyclic peptides are potentially compounds for nitrogen storage or signaling: (**A**) Schizopeptin 791, (**B**) Anabaenopeptin NZ857, (**C**) Nodulapeptin B, and (**D**) Laxaphycin B.

Applying our method to the unlabeled chemical compounds, we created a ranking of the most to least likely secondary metabolites being associated with a diazotrophic cyanobacteria. The top 10 strains are presented in Table 1. The top-ranked strain based on probability of being diazotrophic is IL-208-2-2 from the genus *Schizothrix,* which was isolated in soil. *Schizothrix* in water is known to be diazotrophic [33,34], lending support to this top-ranked strain and this methodology. While the *Schizothrix* genus has NifH sequences in UniProt and is reported in the primary literature [33], the specific strain IL-208-2-2 has no sequences in UniProt, but we have strong evidence to believe it is diazotrophic based on our model and the presence of NifH sequences within the genus. Further below in Table 2 (listed strains) and Table 3 we list the top 10 secondary metabolites based on highest diazotroph association probability. A representative group of these secondary metabolites that are likely diazotroph-associated are visualized in Figure 4, demonstrating a theme of cyclic peptides.

**Table 2:** The strains shown in the table have at least one metabolite with high similarity to known diazotrophs. From the evaluation of strains of known diazotrophic genetics, we would expect these top-ranked strains to be diazotrophs with a high likelihood. The count is how many unique chemicals were present for a given strain. Full results are available in supplemental information.

Strain	Max Probability	Metabolite Count
IL-208-2-2	0.999	1
CCNP1411	0.999	6
CENA352	0.994	2
TAU NZ-3-1	0.986	3
GSV 224	0.984	39
AV1	0.978	17
KAC 11	0.975	4
NIES-81	0.975	2
PCC7310	0.970	1
ITEP-24	0.966	2

**Table 3:** The metabolites shown in the table come from unlabeled data and are in the top 10 in similarity to known diazotroph-associated metabolites. Full results are available in supplemental information.

Compound Name	Predicted Probability
Schizopeptin 791	0.999
Anabaenopeptin NZ857	0.994
Nodulapeptin B	0.978
Nodulapeptin 863	0.977
Nodulapeptin 915a	0.976
Nodulapeptin 855b	0.976
Laxaphycin B	0.974
Laxaphycin B2	0.974
Trichormamide C	0.965
Laxaphycin B3	0.963

### 3.3. Toxicity Is Not Positively Associated with Diazotrophic Secondary Metabolites

**Figure 5:**
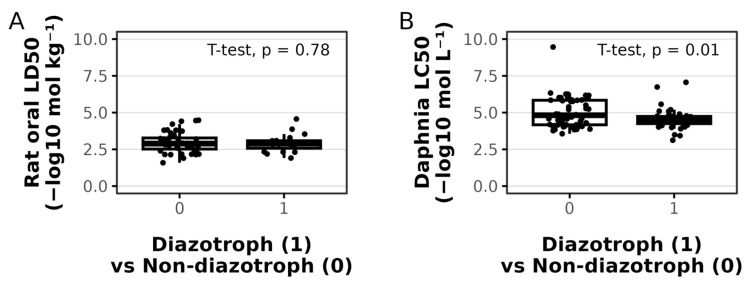
Diazotrophic-associated metabolites are similarly toxic in rats (**A**) and not significantly different in Daphnia magna (**B**) relative to non-diazotrophic-associated metabolites. The LD50 metric of −Log_10_(mol/kg) (or LC50 and its specific metric) is interpreted as a higher value being more toxic.

The ability of secondary metabolites to accurately predict diazotrophic strains raises the question of whether they play a general role across diazotrophic species. One known role of cyanobacterial secondary metabolites is their cytotoxicity towards other organisms. Could there be a difference between the toxicity of diazotrophic- and non-diazotrophic-associated secondary metabolites? Perhaps diazotrophic cells produce more toxic metabolites to fend off predators from consuming them for nitrogen. We ran the labeled metabolites through EPA’s Toxicity Estimation Software Tool [35] for rats and Daphnia magna. Diazotrophic-associated metabolites were not significantly more toxic than non-diazotrophic-associated metabolites as seen below in Figure 5. In rats, the diazotrophic-associated metabolites were of similar toxicity relative to non-diazotrophic metabolites and significantly less toxic than non-diazotrophic metabolites when evaluated by a two-sided *t*-test. While not informative in the function of these diazotrophic-associated metabolites, these results are positive for the use of diazotrophic strains in industrial and agricultural applications, as they seem to not produce more toxic metabolites on average (from the pool of metabolites considered and in the organisms considered). We also did run statistical comparisons of toxicity of labeled metabolites within clusters and again found no significant differences between classes (Supplemental, available in Data Availability link).

## 4. Discussion

Interest in cyanobacterial secondary metabolites for biomedical use cases has been established for decades [35]. However, the systematic and statistical use of cyanobacterial metabolites as indicators for diazotrophy has not been reported prior to this study. We find that chemical structure similarity, as determined by chemical fingerprints, is a strong predictor variable for the likelihood of the producing organisms being diazotrophic. The usefulness of this study is its allowance for predicting and ranking cyanobacterial strains that do not have complete proteomes published as suspected diazotrophs based on their metabolites. This can allow for prioritization of strains to be sequenced and characterized for diazotrophic strength and nitrogenase protection mechanisms.

A closer look at the nine-descriptor heat map (Figure 2A) helps rationalize why chemical similarity is such a strong proxy for diazotrophy. Metabolites from FIX-positive strains are markedly enriched in nitrogen atoms, amide linkages, and carboxyl groups, while showing lower halogen content. Together with the pronounced bias toward cyclic peptide architecture, these features suggest that diazotrophs invest their newly fixed nitrogen into metabolite scaffolds that may act as intracellular nitrogen reservoirs or redox-stable signaling molecules. Unsaturation and ring rigidity (higher DBE values) may further stabilize these peptides against proteolysis, extending their functional lifetime. Hence, the model’s discriminative power stems not from a single diagnostic moiety but from an ensemble of N-dense, macrocyclic traits that collectively define a diazotroph-favored region of secondary metabolite chemical space.

In addition to the practical application of this study, it has also raised questions regarding why diazotrophic cyanobacteria have structurally similar secondary metabolites. Many cyanobacterial secondary metabolites serve to protect the cells from biological threats through various cytotoxic effects. We hypothesized that if diazotroph-associated metabolites served a defensive ecological role, they might exhibit higher toxicity. To examine this, we used the EPA toxicity prediction tool as discussed in the methods to predict acute toxicities (LD_50_ in rat and LC_50_ in Daphnia) of these secondary metabolites.

When we explored the cytotoxicity of secondary metabolites from our training data, no positive association was observed between predicted toxicity level and diazotrophic strains. This is good for the use of diazotrophic cyanobacteria in industrial and agricultural processes but leaves the question open regarding the physiological roles of these metabolites in the diazotrophic cyanobacteria. To temper the meaning of these results, this tool provides an estimate for standard higher organisms and does not account for ecological interactions (like effects on cyanobacterial grazers or competitors specifically). So even if no difference is seen in these general toxicity predictions, it doesn’t completely rule out ecological roles. The purpose of this line of questioning was to explore one sub-hypothesis (toxicity difference between diazotroph and non-diazotroph secondary metabolites), and while useful, it has constraints, and our results from this toxicity question are suggestive rather than conclusive.

It is also worth pointing out constraints to the certainty of this predictive model. One source of potential false-positive predictions is the metabolic promiscuity that characterizes many cyanobacterial secondary-metabolite pathways. A single RiPP-processing enzyme in *Prochlorococcus* MIT9313, for example, can cyclize dozens of distinct peptides, yielding chemically diverse products that are not tied to any specific ecological trait such as nitrogen fixation [36]. Similar promiscuous radical-SAM salvage enzymes have been shown to generate diazotrophy-associated metabolites in *Synechococcus elongatus* [37]. Horizontal gene transfer provides a second, non-exclusive explanation. Plasmid-mediated exchange of NRPS/PKS and other biosynthetic gene clusters is widespread in cyanobacteria, possibly decoupling metabolite production from the essential genetic determinants of nitrogen fixation [38,39]. This means that biosynthetic clusters that are genuinely enriched in diazotrophic donors could be transferred into non-diazotrophic recipients. This could yield metabolite profiles that resemble those of nitrogen-fixers despite the absence of functional *nif* genes, creating false positives in metabolite-only predictions. One more pitfall of this approach is the potential for mis-annotation, such as a false negative (labeling as FIX = 0), because *nif* gene products could hypothetically be missing in incomplete proteomic reporting. We do provide our rationale for a minimum of 100 reported proteins being an acceptable threshold for labeling inclusion in Materials and Methods. Together, enzyme promiscuity, gene cluster mobility, and the potential for mis-annotation constitute systematic sources of potential false positives that chemistry-only models cannot eliminate, narrowing the relative application strength of this model to sample collections or strain isolations where sufficient genetic information on essential nitrogen fixation genes is not present. One final use case of these findings outside of sample prioritization could be remote tracking of cyanobacterial activity if sensitive enough diagnostic tools could be produced.

We note support for the validity of this approach not just in statistical validation, but in practice as evidenced by its positive prediction on unlabeled *Schizothrix* sp. IL-208-2-2. Nitrogen-fixation capability has been demonstrated in non-heterocystous *Schizothrix* isolates where a dominant river-mat strain (MU51) expressed *nifH*, reduced acetylene, and incorporated ^15N_2_ under photo-microaerobic conditions [35]. A subsequent comparative-genomic study confirms that *nif* gene clusters are conserved across sequenced *Schizothrix* genomes [33]. On this basis, we treat *Schizothrix* sp. IL-208-2-2 as putatively diazotrophic, pending direct physiological confirmation.

As more metabolites are discovered in cyanobacteria, the understanding of both the level of conservation and the physiological roles these metabolites play should increase. Our model has shown that secondary metabolites are strongly predictive of diazotrophic genetic complements in cyanobacteria and has opened the question as to why. This also raises the question of if these metabolites are present regardless of if the diazotrophic cyanobacteria is currently expressing that phenotype, which will require further refinement of the available databases. If cyanobacteria express their secondary metabolites regardless of current nitrogen status, this approach could be used to identify diazotrophic strains in the environment even when they are not actively fixing nitrogen.

## 5. Conclusions

We developed an algorithm that predicts the probability that a diazotrophic cyanobacterium is present in a sample based on detected metabolites with high accuracy. We predicted the probability of diazotrophic phenotype in the remaining unlabeled strains of currently undocumented diazotrophic capability from CyanoMetDB and made the predictions available. This will allow interested researchers to prioritize and expedite their exploration of candidate diazotrophic strains. Apart from the practical advances this research has produced, it also raises questions about why certain metabolite structures are predictive of diazotrophic cyanobacteria and how this metabolite-centric approach could be extended to predicting other phenotypes. In respect to the question of why secondary metabolites are predictive of diazotrophs, we investigated one hypothesis and found no statistically positive correlation between secondary metabolite toxicity and diazotrophy. While answering this question is not necessary to act on these predictions and insights, further exploration could lead to an even deeper understanding of cyanobacterial diazotrophy. In future extensions of this study, this metabolite-centric approach could be integrated with genomic or transcriptomic data in future studies to improve predictive power and experimental isolation of predicted diazotrophic strains.

## Figures and Tables

**Table 1:** Multiple different fingerprint approaches had strong predictive performance across metrics. All approaches had rank order capabilities significantly better than random chance (random chance AUC = 0.5, one-sided *t*-test with model greater than random chance hypothesis, significance at *p* < 0.05). These metrics were determined by doing 10 random repeats of 10-fold CV and considering the aggregate of holdout folds from each repeat as a unit of observation. The mean and 95% confidence intervals are given below, and p-values were calculated from these units of observation. Path-based d30 262 k is the fingerprint approach yielding the results referenced throughout this paper.

Fingerprint	Depth	Bits	Accuracy	Precision	Recall	F1	AUC	AUC > Random Chance *p*-Value
PubChem (881 bits)	NA	881	0.905 (0.895–0.914)	0.907 (0.895–0.920)	0.903 (0.894–0.912)	0.905 (0.896–0.914)	0.962 (0.956–0.968)	1.76 × 10^−17^
MACCS 166	NA	166	0.886 (0.875–0.896)	0.897 (0.886–0.908)	0.873 (0.860–0.887)	0.885 (0.874–0.896)	0.957 (0.949–0.966)	4.41 × 10^−16^
Extended d15 262 k	15	262,144	0.874 (0.862–0.887)	0.868 (0.849–0.886)	0.887 (0.875–0.898)	0.877 (0.865–0.888)	0.948 (0.942–0.954)	1.45 × 10^−17^
Path-based d30 262 k (paper)	30	262,144	0.868 (0.854–0.883)	0.874 (0.854–0.894)	0.864 (0.848–0.880)	0.869 (0.855–0.883)	0.950 (0.944–0.956)	1.75 × 10^−17^
Graph 4 k	NA	4096	0.820 (0.809–0.832)	0.866 (0.851–0.882)	0.761 (0.746–0.776)	0.810 (0.797–0.823)	0.864 (0.857–0.871)	4.43 × 10^−16^

## Data Availability

All inputs and outputs are available at https://github.com/jamesyoung93/Secondary-Metabolites-and-Diazotrophs (18 August 2025).

## Supplementary Materials

The full input and output tables are available in the results folder at https://github.com/jamesyoung93/Secondary-Metabolites-and-Diazotrophs (18 August 2025) and https://zenodo.org/records/15778458 (18 August 2025).

## Abbreviations

LOOCV = leave-one-out cross-validation, AUC = area under the (ROC) curve, Cl = abbreviation for cluster, CV = cross-validation, EPA = United States Environmental Protection Agency, F1 = F1 statistical score, FIX = binary label indicating a nitrogen-fixing (diazotrophic) producer (FIX = 1) or non-diazotrophic (FIX =0), LC_50_ = lethal concentration for 50% mortality, LD_50_ = lethal dose for 50% mortality, MDS = multidimensional scaling, NN = nearest neighbor, QSAR = quantitative structure activity relationship.
